# Management of irritable bowel syndrome in primary care: the results of an exploratory randomised controlled trial of mebeverine, methylcellulose, placebo and a self-management website

**DOI:** 10.1186/1471-230X-13-68

**Published:** 2013-04-21

**Authors:** Hazel Everitt, Rona Moss-Morris, Alice Sibelli, Laura Tapp, Nicholas Coleman, Lucy Yardley, Peter Smith, Paul Little

**Affiliations:** 1 Primary Medical Care, School of Medicine, University of Southampton, Southampton, SO17 1BJ, UK; 2 Health Psychology Section, Psychology Department, |Institute of Psychiatry KCL, London Bridge, London, SE1 9RT, UK; 3 Centre for Behavioural Medicine, Department of Practice and Policy, School of Pharmacy, University College London, London, WC1N 1AX, UK; 4 Camborne Physiotherapy Outpatients Department, Camborne Redruth, Community Hospital, Barncoose Terrace, Redruth, England, Cornwall, TR15 3ER, UK; 5 Southampton University Hospital Trust, Southampton, UK; 6 School of Psychology, University of Southampton, Southampton, SO17 1BJ, UK; 7 Southampton Statistical Sciences Research Institute, University of Southampton, Southampton, SO17 1BJ, UK; 8 Primary Medical Care, School of Medicine, University of Southampton, Southampton, SO17 1BJ, UK

**Keywords:** Irritable bowel syndrome, Cognitive behavioural therapy, Web-based intervention, Self-management, Primary care

## Abstract

**Background:**

Many patients with IBS suffer on-going symptoms. The evidence base is poor for IBS drugs but they are widely prescribed and advised in Guidelines. Cognitive Behavioural Therapy (CBT) can be helpful, but availability is poor in the NHS. We developed a web-based CBT self-management programme (Regul8) in partnership with patients and trialled it and common IBS medications in an exploratory factorial RCT to test trial procedures and provide information for a larger trial.

**Methods:**

Patients, 16 to 60 years, with IBS symptoms fulfilling Rome III criteria were recruited via GP practices and randomised to over-encapsulated mebeverine, methylcellulose or placebo for 6 weeks and to 1 of 3 website conditions: Regul8 with a nurse telephone session and email support, Regul8 with minimal email support, or no website.

**Results:**

135 patients recruited from 26 GP practices. Mean IBS SSS score 241.9 (sd 87.7), IBS-QOL 64 (sd 20) at baseline. 91% follow-up at 12 weeks. Mean IBS SSS decreased by 35 points from baseline to 12 weeks. There was no significant difference in IBS SSS or IBS-QOL score between medication or website groups at 12 weeks, or in medication groups at 6 weeks, or IBS-QOL in website groups at 6 weeks. However, IBS SSS at 6 weeks was lower in the No website group than the website groups (IBS SSS no website =162.8 (95% CI 137.4-188.3), website 197.0 (172.4 - 221.7), Website + telephone support 208.0 (183.1-233.0) p = 0.037).

Enablement and Subjects Global Assessment of relief (SGA) were significantly improved in the Regul8 groups compared to the non-website group at 12 weeks (Enablement = 0 in 56.8% of No website group, 18.4% website, 10.5% Website + support, p = 0.001) (SGA; 32.4% responders in No website group, 45.7% website group, 63.2% website + support group, p = 0.035).

**Conclusions:**

This exploratory study demonstrates feasibility and high follow-up rates and provides information for a larger trial. Primary outcomes (IBS SS and IBS QOL) did not reach significance at 6 or 12 weeks, apart from IBS SSS being lower in the no-website group at 6 weeks - this disappeared by 12 weeks. Improved Enablement suggests patients with access to the Regul8 website felt better able to cope with their symptoms than the non-website group. Improved SGA score in the Regul8 groups may indicate some overall improvement not captured on other measures.

**Trial registration:**

ClinicalTrials.gov Identifier (NCT number): NCT00934973.

## Background

Irritable bowel syndrome (IBS) is a common chronic gastrointestinal disorder that affects 10 – 22% of the UK population and costs the National Health Service (NHS) over 200 million pounds a year [1,2]. Abdominal pain, bloating and altered bowel habit affect quality of life, social functioning and time off work [3,4]. Treatment relies on a positive diagnosis, reassurance, lifestyle advice, and drug and psychological therapies [5]. However, many patients suffer on-going symptoms.

Bulking agents and antispasmodics are the most commonly prescribed medications in the UK and Europe. IBS Guidelines [3,5] and a Cochrane review [6] have highlighted the lack of evidence for the current drug management but still recommend them as first line management in primary care. Most studies of anti-spasmodics and bulking agents for IBS were undertaken a long time ago, are of poor quality, with small numbers, in a secondary care setting. Thus, neither doctors nor patients have good evidence to inform prescribing decisions in primary care which is where most patients are managed. However, IBS drugs are recommended in the guidelines [3,5] and very widely used. In 2005, NHS costs were nearly £10 million for mebeverine and over £8 million for fibre-based bulking agents (Prescription Cost Analysis figures). Trials of mebeverine and a fibre-based bulking agent would help to provide evidence to inform prescribing decisions in IBS.

Psychological therapies, such as face to face Cognitive Behavioural Therapy (CBT) have been shown to be helpful for IBS (symptoms and global wellbeing) particularly in the short term, though there is less evidence in the longer term [5,7-11]. NICE Guidelines [5] state that there is good evidence to show a significant global improvement in IBS symptoms with CBT compared to no treatment. NICE recommended psychological therapies for patients with resistant IBS that has not responded to other measures after 12 months. However, availability is very limited, CBT in this format has not been proven to be cost effective [12] and there are problems with high drop-out rates [12]. Web-based CBT has been shown to be helpful for other conditions, e.g. depression [13] and tinnitus [14], insomnia [15] and NICE has recommended the use of web-based CBT for depression, panic and phobia in Primary Care [16]. Thus, web-based CBT could be an efficient, cost effective way of providing help to those with IBS. Despite extensive literature searching before the start of this study, we found no published evaluations of web-based CBT programmes for IBS. A mindfulness study and a small CBT study have since been published [17,18]. Development and testing of a web-based CBT programme for IBS has the potential to make CBT more widely available for IBS with minimally increased costs. The increasing availability of the internet makes this a good medium to provide easily accessible patient information and self-management programmes.

### Aims

To undertake an exploratory factorial RCT to assess the effectiveness of the prescribed medications in UK general practice for IBS: mebeverine (an anti-spasmodic) and methylcellulose (bulking-agent) against placebo, and Regul8, a CBT self-management website for IBS developed specifically for this study.

## Methods

The study protocol has been published on BMC Gastroenterology providing a fuller description of the method [19]. Full Ethical Approval was granted by Southampton A Ethics committee: Reference number: 09/H0502/101.

Patients with IBS were identified by searching GPs’ lists and by opportunistic recruitment of presenting patients. Potential participants were sent a letter inviting them to take part. GP practices in urban and rural settings and differing socio-demographic characteristics were included. Informed written consent was obtained from all participants.

### Inclusion criteria

Patients aged 16 to 60 years with symptoms of IBS that fulfil Rome III criteria (maximum 60 years as NICE guidelines advise that a new change in bowel habit over 60 years should have further investigations [5]) Potentially eligible participants had screening bloods (full blood count (FBC), transglutaminase antibodies (TTG) and a C-Reactive protein (CRP) to exclude alternative diagnoses, i.e. anaemia and Coeliacs disease).

## Exclusion criteria

Atypical symptoms – (unexplained weight loss or rectal bleeding), diagnosis of inflammatory bowel disease, coeliac disease or peptic ulcer disease, pregnant or breast feeding, currently taking or allergy to mebeverine or methylcellulose.

### Regul8 self-management web-based programme

We developed a web-based CBT self-management programme for IBS based on a paper-based manual [20] originally developed and tested by one of our team in a RCT in primary care (RMM) [20]. LifeGuide software was used to develop the website [21]. As each web-based module was developed, ‘Think Aloud’ [22] interviews were undertaken with four patients with IBS to ensure that the website was relevant, understandable, navigable and user friendly. The feedback was used to modify the site during development. The intervention consists of 8 sessions for participants to work through over 6 weeks (see Table 1 for an overview of each session) and includes interactive components such as: development of a personal model, symptom diaries, goal sheets and thought records. Interactive components help users to remember advice, reflect and provide a “substitute” for the therapist [23]. Tailoring feedback to users' symptoms, allows them to focus on personally relevant aspects of the programme. Early in each session participants review key points from the previous session and review their homework, thereby reinforcing previous learning.

**Table 1:** Summary of the Regul8 web-based self-management sessions

**Session 1:** Understanding your IBS	Rationale for self-management which includes the following explanations:
	1. Possible causes of IBS and illustrative physiology of the digestive system together with the functional changes that occur in the gut as a result of IBS.
	2. How the autonomic nervous system (“fight-or-flight” stress system) may interact with the enteric nervous system.
**Session 2:** Assessing your symptoms	Self-assessment of the interaction between thoughts, feeling and behaviours and how these can impact on stress levels and gut symptoms.
	Development of a personal model of IBS which incorporates these elements.
	Homework: Daily diaries of the severity and experience of IBS symptoms in conjunction with stress levels and eating routines/behaviours.
**Session 3:** Managing Symptoms and Eating	Review of the symptom diary.
	Behavioural management of the symptoms of diarrhoea and constipation, and common myths in this area are discussed. Goal setting is explained.
	The importance of healthy, regular eating and not being overly focused on elimination is covered.
	Homework: Goal setting for managing symptoms and regular/healthy eating. Goal setting, monitoring and evaluation continue weekly throughout the programme.
**Session 4:** Exercise and Activity	Importance of exercise in symptom management is covered.
	Identifying activity patterns such as resting too much in response to symptoms or an all-or-nothing style of activity is addressed.
	Homework: Goal setting for regular exercise and managing unhelpful activity patterns if relevant.
**Session 5:** Identifying your thought patterns	Identifying unhelpful thought (negative automatic thoughts) in relation to high personal expectations and IBS symptoms is introduced.
	Link between these thoughts, feelings, behaviours and symptoms is reinforced.
	Homework: Goal setting plus daily thought records of unhelpful thoughts related to personal expectations and patterns of over activity.
**Session 6:** Alternative thoughts	The steps for coming up with alternatives to unhelpful thoughts are covered together with personal examples.
	Homework: Goal setting plus daily thought records including coming up with realistic alternative thoughts.
**Session 7:** Managing Stress and Sleep	Basic stress management and sleep hygiene are discussed.
	Diaphragmatic breathing, progressive muscle relaxation and guided imagery relaxation are presented in video and audio formats.
	Homework: Goal setting for stress management, relaxation techniques and good sleep habits.
**Session 8:** Managing flare-ups and the future	The probability of flare-ups is discussed and patients are encouraged to develop achievable, long term goals and to continue to employ the skills they have learnt throughout the manual to manage flare-ups and ongoing symptoms.

### Intervention

Patients were randomised to: mebeverine (135 mg three times a day), methylcellulose (3 tablets twice a day), or a placebo tablets for 6 weeks. To ensure double blinding, all participants took three over-encapsulated identical tablets in the morning, one at lunchtime and three at dinnertime.

They were also randomised to 1 of 3 website conditions: access to Regul8 with a 30 minute nurse telephone session (to encourage engagement with the CBT programme) and email support on request; the website with minimal support (i.e. technical email support on request), or no website, thus creating 9 groups.

Baseline characteristics were collected by on-line questionnaire including: gender, age, length of symptoms, deprivation score, IBS type and educational level. All patients received a telephone call in the first week to check they had no problems with the medication or paperwork. Those not randomised to website access received standard patient information and were offered website access at the end of the trial.

### Telephone session

The telephone session in the ‘website with telephone support’ group was scheduled after patients completed session 2 where they develop their own personal model of how their thoughts, feelings and behaviours might be contributing to their IBS symptoms. The purpose of the session was for the patients to clarify their model, to help extend their model and to motivate patients to continue using the website. To ensure treatment fidelity one practice nurse delivered all the telephone sessions. Prior to undertaking the sessions, the nurse received six training sessions including a basic introduction to CBT techniques. All telephone sessions were audio-taped. RMM regularly listened to the tapes and provided fortnightly supervision to the nurse during the trial.

### Randomisation

A randomisation list was computer generated independently of the research team. Participants were block randomised in blocks of 9 and stratified by type of IBS (i.e. diarrhoea predominant, constipation predominant, alternating-pattern) as there might be a different response to the medications between these sub-groups.

### Outcome measures

#### Primary outcome measures

Change in the Irritable Bowel Symptom Severity Scale (IBS SSS score) [24] and IBS Quality of Life Questionnaire (IBS-QOL) [25,26] from baseline to 12 weeks. Primary outcome data were collected at baseline and 6 and 12 weeks post randomisation.

The rationale for including two primary outcome measures is that IBS is a chronic relapsing/remitting condition. It is both the severity of the symptoms in themselves and the sufferer’s ability to manage and live with the symptoms and their resultant quality of life that are important. CBT may have a greater effect on peoples’ ability to live with and manage their symptoms than on the severity of the symptoms themselves so it is particularly important to include quality of life as a main outcome in studies such as this.

#### Secondary outcome measures

Patient Enablement Questionnaire [27] (measured at 6 and 12 weeks), Subjects Global Assessment of Relief (SGA of Relief) [28] (measured at 12 weeks), Hospital Anxiety and Depression Scale (HADS) [29] (measured at baseline, 6 and 12 weeks). In SGA of relief participants rate their relief from IBS symptoms on a scale of 1 to 5 ranging from "completely relieved" to "worse". Scores are dichotomized; patients scoring from 1–3 are considered responders, those 4–5, non-responders as has been undertaken in previous studies [20,28]. Patient enablement questionnaire [27] assesses participants’ ability to cope with their illness and life based on 6 questions scored 0 to 2.

Qualitative interviews were undertaken with 30 participants regarding their experience of participating in the trial, the acceptability of the trial procedures, including the medication taking and perceptions of the web-based self-management programme. These results will be presented in a separate paper.

*Sample size:* The aim of this pilot was to have sufficient numbers to assess both the feasibility of trial procedures and to provide an estimate of the likely differences between the groups. The previous trial of CBT in primary care [8] demonstrated a 68 point reduction in symptoms score (a standardised effect size of more than 0.8). Assuming an initial mean symptom score of 250 (SD 80) in all groups, 35 patients were required in each group to detect a difference in score of 53 points (a moderate standardised effects size of 0.66) between the treatment groups (mebeverine, methylcellulose and placebo) or the three website groups (none, minimal support, telephone) for 80% power and 95% confidence. Allowing for drop outs at 20%, we estimated 130 participants would be needed. 135 patients facilitated similar numbers in each of the 9 randomisation groups. Given the different modes of action of the interventions we assumed no interaction between the website and medication interventions.

### Statistical analyses

The statistical analyses were performed with SPSS. Data screening showed that the assumptions of normality were met for the distributions of all the variables except for HADS depression subscale and Enablement scale. Both sub-scales were divided in groups according to their distribution and then ordinal regressions were conducted for analysis. All treatment analyses were intention to treat and considered exploratory. Missing data was handled by the ‘last observation carried forward’ method.

We conducted ANCOVA for the IBS SSS scores, the IBS-QOL scores, and the HADS anxiety sub-scale at 6 and 12 week follow-ups to control for baseline and for potentially confounding variables. We conducted a binary regression for the SGA dichotomous score (Responders/Non responders) at 12 week follow-up.

## Results

### Recruitment

**Figure 1:**
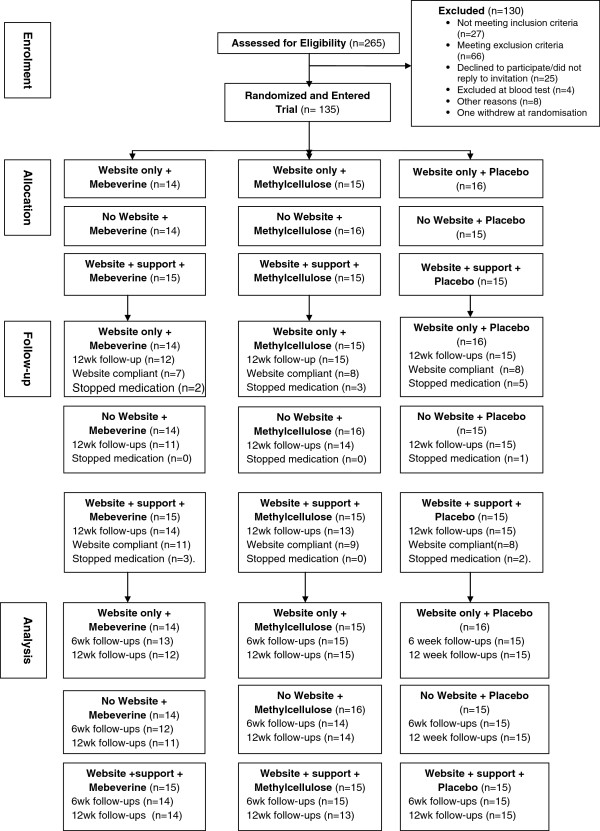
CONSORT diagram.

5715 invitation letters were sent from 26 GP practices to patients on their practice lists with a diagnosis code for IBS. 431 positive replies were received, 265 potential participants completed on-line screening and167 were eligible but 4 of these were excluded after screening blood tests. 135 participants entered the trial (see Figure 1: CONSORT Diagram).

### Decline to participate data

673 patients returned a reply slip declining to take part in the study. Reported reasons for declining: 127 said they did not have time in their daily schedule, 162 did not wish to take medications, 33 were already taking Mebeverine or Methylcelluose and did not wish to stop them, 53 did not wish to take part in the self-management programme, 366 said IBS symptoms had improved and that they did not need additional help, 50 said they did not know they had an IBS diagnosis, and 15 did not have computer access. Patients could choose more than one option for declining.

### Follow–up

A total of 126/135 participants (93%) completed the six week-follow up and 123/135 (91%) completed the twelve week follow-up.

### Compliance with Regul8

91 participants were allocated to either website alone or website with support. The number of sessions undertaken by participants ranged from 0 to 8 (all sessions), with 7 participants completing no sessions and 21 completing 8 sessions, the median was 4 sessions. Compliance was defined as complying with 4 or more sessions. Overall, 51/91 (56%) complied with the self-management programme. 23/45 (51%) participants randomised to website only and 28/46 (61%) randomised to the website plus telephone support complied.

### Compliance with drugs

Compliance with medicine taking overall was 80% based collection of medication boxes at the end of the trial (data available for 91/135). Only 13/135 stopped taking the medications (11%). We had expected some drop out from medication taking considering the requirement to take 7 over-encapsulated tablets daily for six weeks. 7/13 of these participants stated they stopped the medication because their IBS symptoms worsened. 6/13 did not give a reason.

### Telephone sessions

40/46 (87%) participants in the website plus support group received the support telephone session. Of the 6 participants who did not receive the sessions, 3 declined the invitation but continued working on the self-management programme, 1 was not contactable but continued working on the website, and 2 withdrew from the trial after randomisation.

### Stratification by IBS type

This pilot trial showed that it was feasible to stratify participants by IBS type (Constipation, Diarrhoea or Mix pattern IBS) and that trial procedures worked well for this. No statistical analysis by stratification was planned for the pilot to as it was not powered for this. However, a stated aim in the protocol was to examine the data for any worsening of symptoms in the Diarrhoea predominant IBS strata that received methylcellulose. There was no evidence of increased dropouts or reported adverse events, so there is no indication that this group should be excluded from a larger trial.

### Baseline patient characteristics (Tables 2 and 3)

**Table 2:** Randomised participants by website group

**Variable**	**Website only**	**Website with support**	**No website**	**Test**
	**N = 45**	**N = 45**	**N = 45**	
***Age (years), mean (SD)***	45.76 (9.21)	45.13 (10.13)	42.18 (11.48)	F(2,132) = 1.524, p = 0.222
***Length of symptoms (years), mean (SD)***	10.51 (8.19)	12.87 (9.47)	9.00 (8.14)	KW, ap = 0.129
***Median (IQR)***	10.00 (12.00)	12.00 (15.00)	7.50 (8.75)	
***IBS-SSS total, mean (SD)***	237.33 (85.36)	237.30 (100.78)	251.36 (75.92)	F(2,132) = 0.377, p = 0.687
***IBS-QOL score, mean (SD)***	67.32 (17.89)	60.00 (3.13)	64.86 (19.39)	F(2,132) = 1.65, p = 0.20
***HADS score for anxiety, mean (SD)***	8.73 (3.40)	9.46 (3.91)	10.00 (4.12)	F(2,132) = 1.23, p = 0.30
***HADS score for depression, mean (SD)***	4.20 (2.87)	5.26 (3.68)	5.32 (3.56)	KW, ap = 0.263
***Median (IQR)***	4 (4)	5 (6)	5 (5)	
***Deprivation score, mean (SD)***	12.27 (9.07)	8.65 (5.54)	12.81 (9.40)	KW, ap = 0.026*
***Median (IQR)***	10.06 (9.79)	6.57 (7.63)	9.79 (9.21)	
***Gender n (%)***				
***Male***	14 (46.7)	12 (40.0)	4 (13.3)	X^2^(2) = 6.844, p = 0.034*
***Female***	31(29.5)	34 (32.4)	40 (38.1)	
***Type of IBS n (%)***				X^2^(4) = 0.70, p = 0.953
***Constipation***	5 (38.5)	5 (38.5)	3 (23.1)	
***Diarrhoea***	14 (34.1)	13 (31.7)	14 (34.1)	
***Mixed***	26 (32.1)	28 (34.6)	27 (33.3)	
***Severity IBS on IBS SSS n (%)***				
***Mild***	10 (33.3)	12 (40.0)	8 (26.7)	X^2^(4) = 1.37, p = 0.85
***Moderate***	24 (32.4)	23 (31.1)	27 (36.5)	
***Severe***	11 (35.5)	11 (35.5)	9 (29.0)	
***Education n (%)***				X^2^(10) = 3.65, p = 0.97
***No formal***	2 (25.0)	4 (50.0)	2 (25.0)	
***GSCE/O***	11 (31.4)	11 (31.4)	13 (37.1)	
***A level***	11 (32.4)	10 (29.4)	13 (38.2)	
***Degree***	12 (38.7)	10 (32.3)	9 (29.0)	
***Postgraduate***	5 (33.3)	7 (46.7)	3 (20.0)	
***Other***	4 (33.3)	4 (33.3)	4 (33.3)	

**Table 3:** Randomised participants by medicine group

**Variable**	**Mebeverine**	**Methylcellulose**	**Placebo**	**Test**
	**N = 44**	**N = 46**	**N = 46**	
***Age (years), mean (SD)***	45.44 (7.73)	43.52 (12.22)	44.24 (10.57)	F(2,132) = 0.385, p = 0.68
***Length of symptoms(years) - mean (SD)***	11.20 (8.94)	10.30 (9.05)	10.91 (8.34)	KW, ap = 0.783
Median (IQR)	10.00 (11.00)	8.50 (12.00)	10.00 (11.00)	
***IBS-SSS total score - mean (SD)***	270.02 (77.27)	233.15 (89.19)	224.35 (90.79)	F(2,132) = 3.483, p = 0.034*
***IBS-QOL score - mean (SD)***	59.58 (20.60)	65.70 (18.64)	66.51 (19.49)	F(2,132) = 1.65, p = 0.20
***HADS score for anxiety- mean (SD)***	9.23 (4.02)	9.57 (4.19)	9.37 (3.30)	F(2,132) = 0.84, p = 0.92
***HADS score for depression - mean (SD)***	5.76 (3.75)	4.22 (4)	4.93 (3.25)	KW, ap = 0.109
***Median (IQR)***	6.00 (6.00)	3.00 (4.00)	4.00 (6.00)	
***Deprivation score, mean (SD)***	12.19 (8.57)	11.38 (8.87)	10.12 (7.52)	KW, ap = 0.499
***Median (IQR)***	10.06 (9.71)	8.00 (9.56)	8.25 (7.26)	
***Gender - n (%)***				
***Male***	12 (40.0)	9 (30.0)	9 (30.0)	X2(2) = 1.18, p = 0.57
***Female***	31 (29.5)	37 (35.2)	37 (35.2)	
***Type of IBS - n (%)***				
***Constipation***	5 (38.5)	3 (23.1)	5 (38.5)	X2(4) = 0.893, p = 0.932
***Diarrhoea***	13 (31.7)	15 (36.6)	13 (31.7)	
***Mixed***	25 (30.9)	28 (34.6)	28 (34.6)	
***Severity IBSon IBS SSS n (%)***				X2(4) = 11.48, p = 0.021*
***Mild***	3 (10.0)	11 (36.7)	16 (53.3)	
***Moderate***	26 (35.1)	27 (36.5)	21 (28.4)	
***Severe***	14 (45.2)	8 (25.8)	9 (29.0)	
***Education - n (%)***				
***No formal***	4 (50)	3 (37.5)	1 (12.5)	X2(10) = 5.67, p = 0.85
***GSCE/O***	12 (34.3)	9 (25.7)	14 (40)	
***A level***	10 (29.4)	12 (35.3)	12 (35.3)	
***Degree***	8 (25.8)	11 (35.5)	12 (38.7)	
***Postgraduate***	6 (40.0)	5 (33.3)	4 (26.7)	
***Other***	3 (25.0)	6 (50.0)	3 (25)	

The mean age of participants was 44 years, 78% were women. The mean length of symptoms reported was 10.8 years (SD = 8.7). Mean IBS-SSS score at baseline was 241.9 (SD = 87.7), which represents a moderate severity of IBS. Baseline characteristics were similar across the groups (please see Tables 2 and 3), with the exception of Gender (X^2^ p = 0.034) and Deprivation scores (KW p = 0.026) in the website conditions and IBS-SSS total mean (Fp = 0.034) (also reflected in the symptom severity classification (p = 0.021)) in the drug conditions. We assessed these variables in the ANCOVA/regression analyses. However, none were significant, so they were not included in the models.

### Primary outcomes

**Figure 2:**
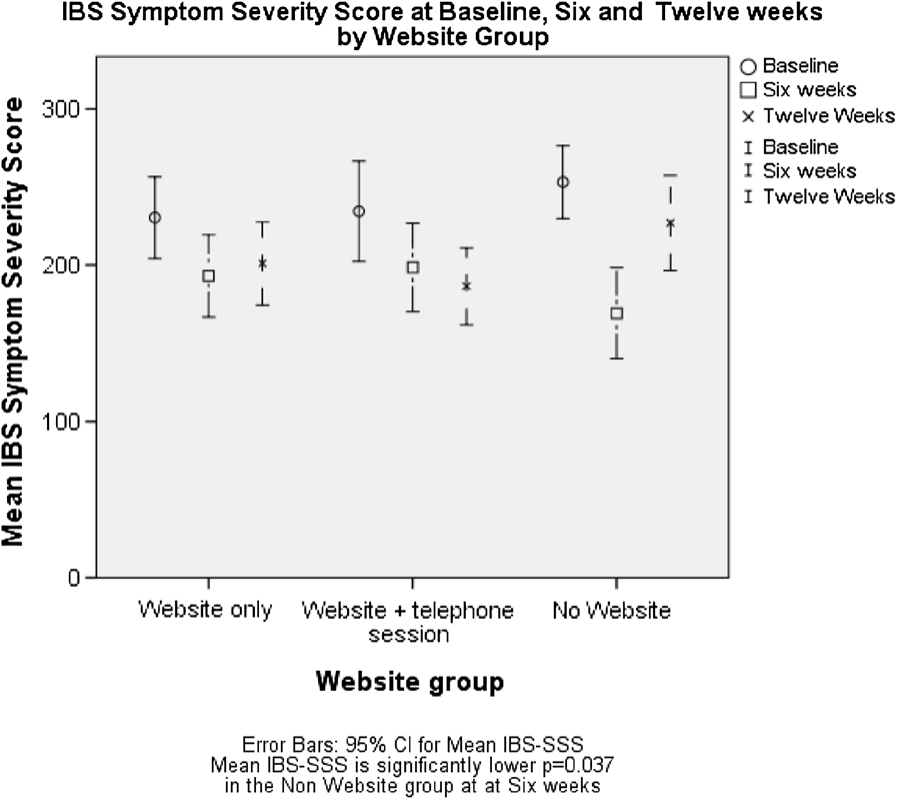
IBS symptom severity score at baseline, six and twelve weeks by website group.

**Figure 3:**
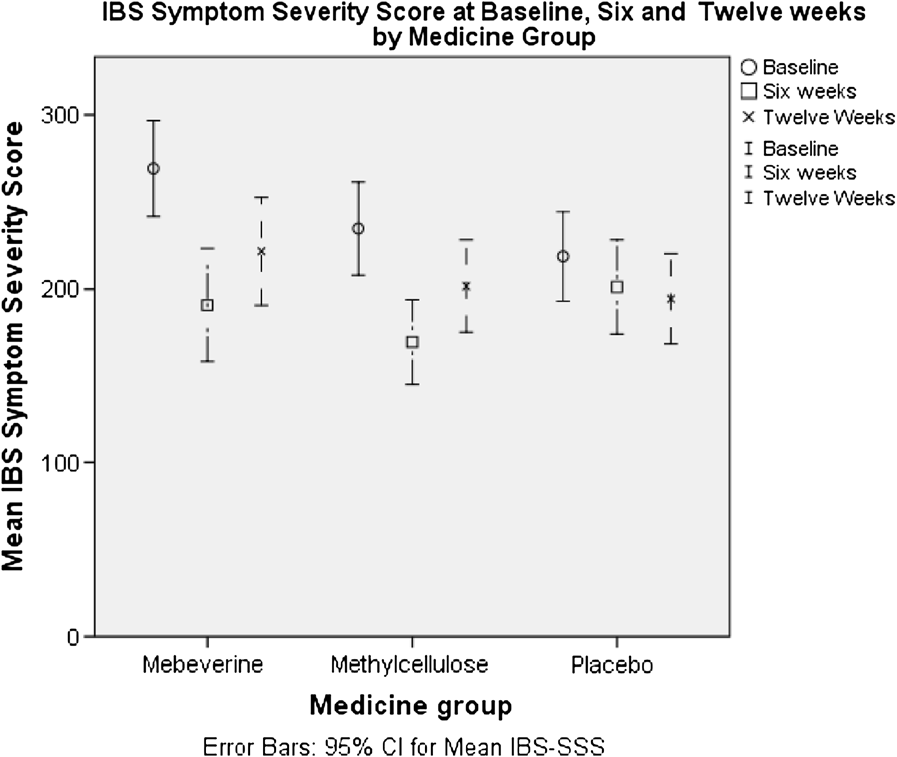
IBS symptom severity score at baseline, six and twelve weeks by medicine group.

The mean of the IBS-SSS score of the whole sample decreased (positive change) 52 points from baseline to the 6 week point and 35 points from baseline to the 12 week point. The mean of the IBS-QOL score of the sample increased (positive change) 6 points from baseline to the 6 week point and 5 points from baseline to the 12 week point (Figures 2 and 3).

At 6 weeks there was no significant difference in IBS SSS or IBS-QOL between the medicine groups or for IBS-QOL for the website groups (see Tables 4 and 5). However, there was a statistically significant difference seen in the IBS SSS for the website, with the no website group having a lower IBS SSS score than the website groups (IBS SSS no website =162.8 (95% CI 137.4-188.3), website 197.0 (172.4 - 221.7), Website + telephone support 208.0 (183.1-233.0) p = 0.037). This difference disappeared by 12 weeks (see Figure 2 and Tables 4 and 6).

**Table 4:** Main and secondary outcomes for website groups at six week follow up

**Variable**	**Website**	**Website + support**	**No website group**	**Significance**
***IBS-SSS total mean (n = 123)***	197.0 (172.4 - 221.7)	208.0 (188.1 - 233.0)	162.8 (137.4 -188.3)	p = 0.037*
***IBS-QOL score (n = 123)***	71.6 (67.9-75.2)	67.6 (63.9-71.3)	69.4 (65.7-73.1)	p = 0.315
***HADS score for anxiety (n = 114)***	8.9 (7.9-9.8)	9.7 (8.8-10.6)	8.3 (7.3-9.2)	p = 0.109
***HADS score for depression (n = 114) (%)***				p = 0.122
*Normal (0–7)*	35 (89.7)	28 (71.8)	31 (79.5)	
*Mild (8–10)*	2 (5.1)	8 (20.5)	8 (20.5)	
*Moderate or Severe (11–21)*	2 (5.1)	3 (7.7)	0 (0)	
***Enablement score (n = 113) (%)***				p = 0.000**
*0*	7 (18.4)	4 (10.5)	21 (56.8)	
*1-2*	7 (18.4)	5 (13.2)	4 (10.8)	
*3-5*	13 (34.2)	15 (39.5)	7 (18.9)	
*6-12*	11 (28.9)	14 (36.8)	5 (13.5)	

Analysis of the 6 week follow-up scores.

ANCOVA for IBS SSS, IBS-QOL and HADs Anxiety, Mean (95% CI). Ordinal Regression for HADs Depression and Enablement (n (%).

**Table 5:** Main and secondary outcomes for medicine groups at six week follow up

**Variable**	**Mebeverine**	**Methylcellulose**	**Placebo**	**Significance**
***IBS-SSS total mean (n = 123)***	183.3 (156.9-209.7)	174.0 (149.7-198.4)	210.6 (185.6-235.5)	p = 0.106
***IBS-QOL score (n = 123)***	69.5 (65.7-73.4)	69.4 (65.8-73.0)	69.7 (66.0-73.3)	p = 0.994
***HADS score for anxiety (n = 114)***	8.7 (7.7-9.7)	9.0 (8.1-9.9)	9.2 (8.3-10.1)	p = 0.743
***HADS score for depression (n = 114) (%)***				p = 0.590
*Normal (0–7)*	29 (30.9)	36 (38.3)	29 (30.9)	
*Mild (8–10)*	3 (16.7)	5 (27.8)	10 (55.6)	
*Moderate or Severe (11–21)*	2 (40.0)	0 (0)	3 (60.0)	
***Enablement score (n = 113)***				p = 0.563
*0*	6 (19.4)	14 (35.9)	12 (27.9)	
*1-2*	5 (16.1)	6 (15.4)	5 (11.6)	
*3-5*	9 (29.0)	11 (28.2)	15 (34.9)	
*6-12*	11 (35.5)	8 (20.5)	11 (25.6)	

Analysis of the 6 week follow-up scores.

ANCOVA for IBS SSS, IBS-QOL and HADs Anxiety, Mean (95% CI). Ordinal Regression for HADs Depression and Enablement (n (%).

**Table 6:** Main and secondary outcomes for website groups at twelve week follow up

**Variable**	**Website**	**Website** + **support**	**No website group**	**Significance**
***IBS-SSS total mean (n = 123)***	207.9 (187.6 – 228.1)	193.4 (173.0- 213.8)	218.2 (197.4-238.9)	p = 0.243
***IBS-QOL score (n = 123)***	71.6 (67.2 - 76.1)	69.5 (65.0 - 74.0)	64.3 (59.8 – 68.9)	p = 0.068
***HADS score for anxiety (n = 114)***	8.8 (8.0- 9.7)	9.2 (8.3 -10.0)	8.0 (7.1 -8.8)	p = 0.123
***HADS score for depression (n = 114) (%)***				p = 0.981
*Normal (0–7)*	32 (84.2)	31 (79.5)	30 (81.1)	
*Mild (8–10)*	6 (15.8)	4 (10.3)	4 (10.8)	
*Moderate or Severe (11–21)*	0 (0)	4 (10.3)	3 (8.1)	
***Enablement score (n = 113) (%)***				p = 0.000**
*0*	7 (18.4)	4 (10.5)	21 (56.8)	
*1-2*	7 (18.4)	5 (13.2)	4 (10.8)	
*3-5*	13 (34.2)	15 (39.5)	7 (18.9)	
*6-12*	11 (28.9)	14 (36.8)	5 (13.5)	
***SGA (%)***				
*Responders*	16 (45.7)	24 (63.2)	12 (32.4)	p = 0.035*
*Non responders*	19 (54.3)	14 (36.8)	25 (67.6)	

Analysis of the 12 week follow-up scores.

ANCOVA for IBS SSS, IBS-QOL and HADs Anxiety, Mean (95% CI). Ordinal Regression for HADs Depression, Enablement and SGA (n (%).

No significant difference was found between the self-management website groups or the medicine groups for the IBS SSS or quality of life at the twelve week follow up. (see Tables 6 and 7 for summary statistics).

**Table 7:** Main and secondary outcomes for medicine groups at twelve week follow up

**Variable**	**Mebeverine**	**Methylcellulose**	**Placebo**	**Significance**
***IBS-SSS total mean (n = 123)***	203.1(181.3- 223.0)	205.4 (185.3 -225.5)	210.9 (191.1 - 230.7)	p = 0.865
***IBS-QOL score (n = 123)***	68.9 (64.1 – 73.6)	66.3 (61.9 – 70.8)	70.2 (65.9 – 74.6)	P = 0.453
***HADS score for anxiety (n = 114)***	8.2 (7.3 – 9.1)	8.7 (7.9- 9.6)	9.0 (8.2 -9.8)	p = 0.422
***HADS score for depression (n = 114) (%)***				P = 0.655
*Normal (0–7)*	25 (78.1)	35 (89.7)	33 (76.7)	
*Mild (8–10)*	5 (15.6)	2 (5.1)	7 (16.3)	
*Moderate or Severe (11–21)*	2 (6.3)	2 (5.1)	3 (7.0)	
***Enablement score (n = 113)***				P = 0.231
*0*	6 (19.4)	14 (35.9)	12 (27.9)	
*1-2*	5 (16.1)	6 (15.4)	5 (11.6)	
*3-5*	9 (29.0)	11 (28.2)	15 (34.9)	
*6-12*	11 (35.5)	8 (20.5)	11(25.6)	
***SGA (%)***				
***Responders***	16 (57.1)	17 (43.6)	19 (44.2)	P = 0.538
***Non responders***	12 (42.9)	22 (56.4)	24 (55.8)	

ANCOVA for IBS SSS, IBS-QOL and HADs Anxiety, Mean (95% CI). Ordinal Regression for HADs Depression, Enablement and SGA (n (%).

There was no evidence of interactions between the medicine and website groups in any of the analyses.

The mean scores suggest improvements across all groups particularly at the 6 week (end of treatment) period (Tables 4 and 5). There was however a trend for continued improvement in the self-management groups (particularly those who had the telephone support) whilst those in the no website group and the medication groups appeared to lose some of these gains at 12 weeks (see Figures 2 and 3 and Tables 6 and 7).

### Secondary outcomes

No significant difference was found in HADs, Enablement or SGA between the medication groups or in the HADs scores between the website groups (Tables 4 and 7). A highly significant difference was found in the Enablement scores at 6 and 12 week follow-up between the website conditions and the control group (Tables 4 and 6). The participants in the Website groups reported better Enablement than the ones in the no website group. The higher scores were found in the Website plus support condition. This suggests that the participants who had access to the website coped better with their IBS symptoms than the control group.

A significant difference was also found in the SGA at 12 week follow-up between the website groups (Table 6). The participants in the website groups had better scores than the no website group, indicating that the participants who had access to the website rated their overall IBS symptom relief as significantly better than the individuals who had no access to the website.

## Discussion

### Summary of main findings

The trial has demonstrated that it is feasible to recruit and randomise patients with IBS from many GP practices to a complex trial and achieve high follow-up rates.

The results must be interpreted with caution as this is an exploratory study with relatively low numbers and power. No statistically significant differences were seen between the medication groups for the main outcome measures (IBS SSS and IBS-QOL) at 6 or 12 weeks. Nor were there any statistically significant differences between the website groups for the IBS-QOL at 6 or 12 weeks or IBS SSS at 12 weeks. However, there was a statistically significant difference seen in the IBS SSS for the website at 6 weeks, with the ‘no website’ group having a lower IBS SSS score than the website groups, but this difference disappeared by 12 weeks.

There were no differences in the secondary outcomes in the medicine groups.

In the website groups, there was significantly increased Enablement at 6 and 12 weeks and a significantly more participants scored Subjective Assessment of Global relief (SGA) as improved at 12 weeks compared to the No Website group.

### Strengths and limitations

This exploratory trial was the first to look at providing CBT as a web-based self-management programme for patients with IBS. The factorial design allowed the feasibility of undertaking a trial to assess the effectiveness of common IBS medications to be explored at the same time.

Participants were asked to take 7 large over-encapsulated tablets a day for 6 weeks. Despite this, compliance with the medication taking was good.

The lack of difference between the medication groups may be due to low power with the limited numbers in this trial. A larger trial will be able to provide more definitive results. Ideally, smaller trial medication would be sourced for a larger trial to further improve compliance and reduce the placebo effect of taking the tablets.

Expectancy effects (i.e. people's expectancy of web treatment and drug treatment) and the placebo effect may have influenced the trial results. We did not measure treatment expectancy effects in this study.

Over 5700 patients were invited by their GP to participate in this study with 135 being randomised. Thus a small subset of the patients recorded as having IBS by their GPs entered the study. This low recruitment rate is not unexpected in a study requiring patients to be on no initial medication, to take many large tablets a day and participate in a self-management programme for 6 weeks, but it may reflect a more committed group of participants and affect the generalisability of the study findings. Reasons for declining to enter the study have been sought and recorded and the study sample is similar in gender and age to the invited participants.

### Comparisons with other studies

There are a limited number of published trials of CBT for IBS but they indicate a positive effect for CBT on IBS over a range of outcome measures [5,9,18,30] in the short-term (up to 3 months). However, information is lacking for longer term follow up. For instance, a trial of face to face CBT plus mebeverine (6 × 50 minute CBT sessions over 6 week delivered by general practice nurses) against mebeverine alone in a primary care setting in London [8] showed a significant reduction in IBS-SSS in the CBT arm at 3 months but not at 12 months follow up.

The lack of improvement in the IBS SSS and IBS-QOL in the website self-management groups in this study is disappointing as the paper-based self-management programme from which the Regul8 website was developed [20] did show a significant improvement in IBS SSS scores in the self-management group compared to the control group. It may be that an effect of the website was masked by the general improvement in all groups during the length of the trial. It is acknowledged in many trials that IBS symptoms have a large placebo response to any intervention and this can make it difficult to distinguish true treatment effects, particularly in a small study. However, we found a trend for continued improvement in the self-management group (particularly those who had the telephone support) whilst those in the No Website group and the medication groups appeared to lose some of these gains at 12 weeks (see Figures 2 and 3). Longer term follow up may reveal differences between the website groups. Two other reasons why the Regul8 programme may be less successful than the paper-based manual are that: the earlier study used an experienced therapist, and had significantly more therapist contact time. In this study a nurse with only minimal CBT training delivered the intervention. In addition, of those that had access to the Regul8 programme, half had no therapist support at all and half had one, 30 min telephone support session, whereas in the trial of the paper-based manual, one face to face and two telephone support sessions were offered to participants (3 hours in total). Therapist support is expensive compared to a stand-alone website and thus was deliberately kept to a minimum for this trial. However, recently published trials of internet interventions for IBS [18,30] and qualitative data from this study suggest that it is important to assist engagement with the website.

The finding that Enablement scores were higher in those that had access to the Regul8 programme suggests that these participants felt better able to cope with their symptoms. Additionally, those that received the telephone session had higher Enablement than those with access to the website without support. This suggests benefit from the website which is enhanced by therapist support and strengthens the postulation that the amount of support offered in this study may have been insufficient. Ability to cope with IBS symptoms and continue a full life is clearly an important outcome irrespective of symptom severity. Improvements in the Work and Social Adjustment Scale, a measure of the ability to continue work and other activities with IBS, was shown to be improved by CBT in the long term (12 month follow up) in the Kennedy trial [8].

The lack of any significant effect on IBS outcomes in the medication arms of this study, may be due to limited power, and would need to be confirmed in a larger trial but are not unsurprising as a Cochrane review [6] has highlighted the poor evidence of their effectiveness.

### Implications for future research and clinical practice

The factorial trial design for this study had the benefit of providing data for both the medication and the website groups but this may have limited the ability of the trial to show an effect for the CBT self-management programme, since the key to self-management is being able to attribute symptom improvement to cognitive and behavioural changes. In a blinded trial participants would have had some difficulty knowing whether any change in their symptoms was due to the medication they were taking or to the CBT self-management principles they were trying to employ. This issue was raised in discussion with patients in the qualitative interviews. The effect of including medication the factorial design, may have been to reduce their confidence in employing the CBT techniques. Thus a definitive trial of the website should probably not include a blinded medication component.

When recruiting participants from primary care for this trial, it was found to be important to screen potential participants with the ROME III and exclusion criteria as 98 of the 265 potential participants who completed the screening questionnaire either did not fulfil the ROME criteria or met exclusion criteria.

## Conclusion

This study demonstrates that a trial of this type is feasible in primary care and can be successfully completed with robust trial procedures and high levels of follow up. A larger trial (or trials) would have the power to determine more definitive outcomes. Undertaking separate medicine and website self-management programme trials is likely to be appropriate in the larger trials. Increasing the therapist support sessions for the web-based self-management programme would seem logical approach to try to further increase patient enablement, which is an important aspect of self-managing a chronic condition such as IBS.

## Competing interests

The authors declare that they have no competing interests.

## Authors’ contributions

HE, RMM, PL and LY were involved in the conception and design of the study and applied for funding. HE wrote the first draft of the grant application, is Lead investigator and Principal Investigator. RMM oversaw the adaptation of the paper-based CBT manual into the website, the development of the telephone protocol, and nurse training and supervision. LT and AS developed the website and managed the trial. LY oversaw the website programming package. NC (Consultant Gastroenterologist) was a clinical expert advisor. PS developed the Statistical Plan and Randomisation. All Authors read and approved the final manuscript.

## Pre-publication history

The pre-publication history for this paper can be accessed here:

http://www.biomedcentral.com/1471-230X/13/68/prepub
